# The Clinical Spectrum of Adrenoleukodystrophy at a Portuguese Tertiary Hospital: Case Series and Review of Literature

**DOI:** 10.7759/cureus.52496

**Published:** 2024-01-18

**Authors:** Catarina Menezes, Ana Losa, Sara Mosca, Ana de Carvalho Vaz, Catarina M Figueiredo, Cristina Garrido, Teresa Borges, Joana Borges Correia

**Affiliations:** 1 Pediatrics, Centro Materno Infantil do Norte - Centro Hospitalar Universitário de Santo António, Porto, PRT; 2 Pediatric Endocrinology, Centro Hospitalar Entre Douro e Vouga, Santa Maria da Feira, PRT; 3 Pediatric Neurology, Centro Materno Infantil do Norte - Centro Hospitalar Universitário de Santo António, Porto, PRT; 4 Pediatric Endocrinology, Centro Materno Infantil do Norte - Centro Hospitalar Universitário de Santo António, Porto, PRT; 5 Pediatrics, Reference Centre for Metabolic Disorders, Centro Materno Infantil do Norte - Centro Hospitalar Universitário de Santo António, Porto, PRT

**Keywords:** peroxisomal disorders, demyelinating disorders, cerebral x-linked adrenoleukodystrophy, x- linked adrenoleukodystrophy, adrenal insufficiency (ai)

## Abstract

Adrenoleukodystrophy, a rare genetic disease associated with the X chromosome (X-ALD - X-linked adrenoleukodystrophy), predominantly affects males and stems from mutations in the *ABCD1* gene, responsible for transporting very long chain fatty acids (VLCFA) into peroxisomes. It leads to adrenal insufficiency (AI) and axonal demyelination. In males, the phenotype varies from isolated adrenocortical insufficiency and progressive myelopathy to cerebral adrenoleukodystrophy (CALD). The aim of this case series is to characterize patients with different clinical presentations of X-ALD with follow-up at a tertiary Portuguese hospital.

All four patients were males, and the median age at the diagnosis was 5 years. Three patients were diagnosed through family screening, with the oldest already displaying hyperpigmentation. Two distinct forms were identified: adolescent CALD (25%) and isolated primary adrenal insufficiency (75%). Analytical studies revealed elevated plasma VLCFA levels in all cases, and genetic analysis demonstrated two different mutations in the *ABCD1* gene.

This disorder requires early diagnosis for improved prognosis. Screening male children with primary AIfor X-ALD using a VLCFA panel should be considered, particularly after ruling out the most common causes or when learning difficulties are evident. Genetic confirmation of the diagnosis is essential, enabling genetic counseling, family planning, and preimplantation genetic diagnosis.

## Introduction

Adrenal insufficiency (AI) is a potentially fatal disease resulting from a compromised release of adrenal glucocorticoid and mineralocorticoid hormones. Congenital adrenal hyperplasia (CAH) is the most common cause [1,2]. Nonetheless, in a child with possible AI, there are other causes that should be considered, including autoimmune disorders and metabolic causes.

One of the less common causes (Borchers et al [3] reported findings in 6% of cases) is X-linked Adrenoleukodystrophy (X-ALD), a rare genetic disorder that affects primarily males. It results from mutations in the *ABCD1 *gene located on the long arm of the X chromosome, Xq28. The function of this gene is to facilitate the transportation of very long chain fatty acids (VLCFA) into peroxisomes to promote their degradation by oxidation. The accumulation of VLCFA in tissues and body fluids leads to adrenal insufficiency and axonal demyelination [4-6]. Currently, the exact pathophysiology of X-ALD is still unknown, but some hypotheses are described in the literature, namely neurotoxicity due to VLCFA accumulation, causing cell membrane instability and oxidative stress [4,7]. On the other hand, there is a preferential accumulation of VLCFA in the adrenal cortex (mostly in zona reticularis and zona fasciculata), and for this reason, the adrenal manifestations of X-ALD tend to be more primary cortisol insufficiency and androgen deficiency. Adrenocorticotropic hormone (ACTH) levels exceeding 100 pg/mL and cortisol levels below 5-10 mcg/dL are suggestive of primary adrenal insufficiency [8]. Furthermore, the VLCFA may also be integrated into the lipid cell membranes and interfere with ACTH and gonadotropins binding to their receptors [1]. 

The most frequent manifestations include learning and behavioral problems, cutaneous hyperpigmentation and/or muscle weakness. Nevertheless, the range of clinical presentations in males with X-ALD spans from asymptomatic cases and isolated adrenocortical insufficiency to progressive myelopathy and cerebral demyelination (CALD), with no established correlation between genotype-phenotype [2,4,9,10]. The definitive diagnosis is suggested by high serum levels of VLCFA and confirmed through genetic assessment. Although multiple treatment options have been reported, hematopoietic stem cell transplantation (HSCT) is still the first-line and most effective therapy for X-ALD patients with cerebral involvement. Nonetheless, several studies have assessed a new treatment with the transplantation of autologous, genetically modified hematopoietic stem and progenitor cells (HSPC) transduced with lentiviral vectors. This option would be fundamental to overcoming some of the immunological complications of HSCT, namely, graft-versus-host disease [8,11].

The prognostic is generally poor, as patients may die within a few years after being diagnosed or live with several health-related problems. Reaching an early and correct diagnosis is critical to assessing potential associated comorbidities, initiating long-term management, and providing genetic counseling.

The aim of this case series is to analyze the clinical spectrum of patients with X-ALD and study their follow-up at a Portuguese tertiary care hospital.

## Case presentation

We present four patients with X-ALD, all male, from two different families, and with a median (25th-75th percentile, P25-P75) age at the diagnosis of 5 (3-12.5) years. The definitive diagnosis in all cases was achieved between March 2019 and December 2022.

Family 1

The first family concerns a young woman (30 years old) who was confirmed to be an *ACBD1 *gene mutation carrier following the death of her two eldest brothers at a young age. The older brother presented at the age of seven years old with visual impairment, followed by muscle weakness and paralysis at 10 years old. He was diagnosed after performing a brain magnetic resonance imaging (MRI), which showed white matter lesions suggestive of CALD and elevated levels of VLCFA; he died at the age of 27 years. The second brother was diagnosed after a family screening and died at 10 years of age with progressive nervous system deterioration.

Case 1

Our first patient is the woman's son, 17 months old, who was diagnosed with X-ALD after a family screening. At his initial evaluation, he had a normal psychomotor development with no signs of AI. During his follow-up, he presented with a development delay unrelated to the disease, as his brain MRI remained normal. At that moment, he had elevated ACTH levels (118 pg/mL; normal range 9-52 pg/mL) with low cortisol (6.3 mcg/dL; normal range >10 mcg/dL); a cosyntropin stimulation test was performed (9.8 mcg/dL at 60’ - cortisol response <18 μg/mL is diagnostic of glucocorticoid deficiency) and supported the diagnosis of isolated AI. Currently, he has a multidisciplinary follow-up (Pediatric Endocrinology, Neurology, and Metabolic Diseases) and is under hydrocortisone treatment (10.7 mg/m2/day three times a day, oral).

Family 2

Case 2

**Figure 1:**
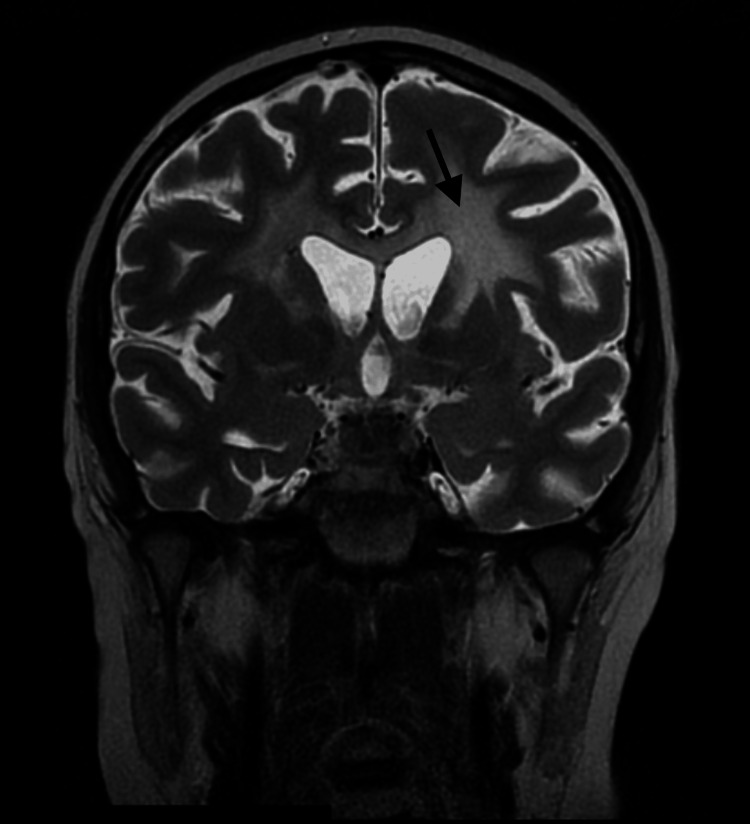
Brain MRI (T2-weighted coronal image) showing subcortical white matter hyperintensity. MRI: Magnetic Resonance Imaging

**Figure 2:**
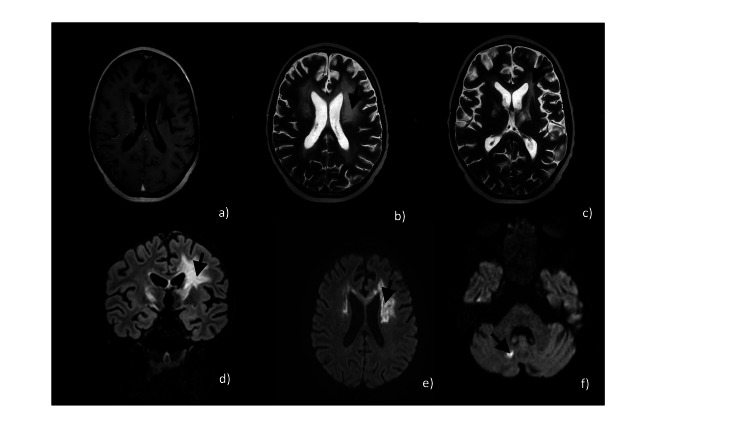
Hypointense area in T1 axial image post-contrast (a); hyperintense area in T2 ((b) and (c)) and T2-FLAIR (d) axial images without diffusion restriction ((e) and (f)); and without peripheral contrast enhancement (a), located in the cerebral and cerebellar white matter. FLAIR: Fluid-attenuated inversion recovery

The next three patients are brothers. The second case (middle brother) had his initial manifestation (metabolic acidosis with persistent vomiting and ketotic hypoglycemia - consistent with an adrenal crisis) at the age of six and began treatment with hydrocortisone (10 mg/m2/day three times a day, oral) and fludrocortisone (0.1 mg/day, once a day, oral). However, the definitive diagnosis of X-ALD was only achieved after presenting additional learning and behavioral problems at 11 years old. His brain MRI revealed posterior and anterior white matter patterns (including cerebellar changes) that were consistent with CALD (Figure 1 and Figure 2) and had positive VLCFA. After the diagnosis, he was sent to the Transplantation Center, where he was submitted to an HSCT (fully matched unrelated donor), about eight months ago. Since then, he has been admitted several times due to HSCT-related complications and is now waiting for clinical re-evaluation to check the evolution of the primary disease. 

Case 3

The third case pertains to the older brother (14 years old), who was evaluated subsequent to his brother's diagnosis. He had a normal neurocognitive development, but he already presented increased skin hyperpigmentation since the age of four years old and had an episode of adrenal crisis with hyponatremia and metabolic acidosis at 6 years. His brain MRI was normal. Laboratory findings included high VLCFA serum levels and marked elevation (8050 pg/mL; normal range 9-52 pg/mL) of ACTH levels and low cortisol levels (3.8 mcg/dL; normal range >5-10 mcg/dL). A therapeutic regimen with hydrocortisone (9.8 mg/m2/day three times a day, oral) was instituted and he presented a significant clinical improvement and still has no neurological involvement.

Case 4

The last case belongs to the younger brother (five years old). An early childhood intervention was started due to his hyperactivity and speech delay when he was four years old. He had a slightly increased skin hyperpigmentation and altered left cutaneous plantar reflex at his first evaluation. His brain MRI was unremarkable. High levels of VLCFA were present, just like the older brothers, and a moderate elevation of ACTH values (690 pg/mL) with a cortisol of 9.1 mcg/dL. Since the clinical findings were mild and the biochemical results were not completely clear, a cosyntropin stimulation test was performed (7.1 mcg/dL at 60’), which supported the diagnosis of isolated AI. The boy was then treated with hydrocortisone (9.23 mg/m2/day three times a day, oral) with partial clinical improvement and is currently under brain MRI screening every 6 months.

This study reported two variations of the disease - adolescent CALD with AI (one case) and isolated primary AI (three cases). The genetic assessment (through *ABCD1 *gene sequencing) was performed in all patients revealing two different mutations in the *ABCD1 *gene (hemizygous mutation in c.1850G>A (p.R617H) exon 8 and c.1415_1416delAG(p.Q472fs*83) exon 5), which together with increased VLCFA levels confirmed the X-ALD diagnosis. Currently, all patients keep follow-up at the Pediatric Endocrinology Unit, under treatment with hydrocortisone, and one patient also with fludrocortisone.

Table 1 summarizes the phenotypes, age of presentation, brain MRI findings, treatment, and genetic testing results. 

**Table 1:** Summarizing phenotypes, age of presentation, brain MRI findings, treatment, and genetic testing results. MRI: Magnetic Resonance Imaging; CALD: cerebral adrenoleukodystrophy; AI: adrenal insufficiency

	Family 1	Family 2		
	Case 1	Case 2	Case 3	Case 4
Age of presentation	17-months-old	6-year-old	14-year-old	5-year-old
Phenotype	Isolated AI	CALD	Isolated AI	Isolated AI
Brain MRI findings	Normal	White matter pattern consistent with CALD (Figures 1, 2)	Normal	Normal
Treatment	Hydrocortisone 10,7mg/m2/day (three times a day, oral)	Hydrocortisone 10mg/m2/day (three times a day, oral) and Fludrocortisone 0,1 mg/day (once a day, oral)	Hydrocortisone 9.8 mg/m2/day (three times a day, oral)	Hydrocortisone 9,23mg/m2/day (three times a day, oral)
Genetic testing results	Mutation in *ABCD1 *gene (hemizygous mutation in c.1850G>A (p.R617H) exon 8)	Mutation in *ABCD1 *gene (hemizygous mutation inc.1415_1416delAG (p.Q472fs*83) exon 5)	Mutation in *ABCD1 *gene (hemizygous mutation inc.1415_1416delAG(p.Q472fs*83) exon 5)	Mutation in *ABCD1 *gene (hemizygous mutation in c.1415_1416delAG (p.Q472fs*83) exon 5)

## Discussion

X-ALD is a disorder that requires early detection. The clinical manifestation of ALD varies widely, and without clinical suspicion, it is likely to progress before being diagnosed [12]. The three most common forms of presentation include: isolated AI (peak incidence from 3 to 10 years), cerebral demyelination (usually between 4-12 years old, peak onset at 7 years), and progressive myelopathy (most frequently around 20-40 years, but median age of 28 years old) [4-6,8]. Isolated AI presents with weight loss, vomiting, fatigue, and increased skin hyperpigmentation; progressive myelopathy’s manifestations include spastic paraparesis, sensory ataxia, bladder or sexual impairment, and possible intact cognitive function; and, finally, CALD presents with muscle weakness, hyperactivity, learning disabilities, hearing and visual impairment, swallowing difficulties and progressive nervous system deterioration (decreased fine motor control, paralysis and coma) [1,2,7,13,14].

Furlan et al [4] reported that CALD was the most common phenotype (approximately 45%), with a mean age at diagnosis of seven years old and whose first symptom was learning difficulties. Jiang et al. stated that the main sign in the first manifestation was skin hyperpigmentation, which agrees with what was found in our sample, as isolated AI was the most prevalent phenotype [16]. These forms of presentation may vary during the disease, with the myelopathy form being able to progress to CALD or patients with isolated AI also beginning to have some associated neurological dysfunction [4,8]. One of the possible explanations for the absence of myelopathy cases in this sample is its higher prevalence in young adults (18 years old or older, thus not a pediatric patient). In this age group, it usually manifests with spastic paraparesis, sensory ataxia, and compromised sphincter control. Peripheral neuropathy may be the first manifestation, and sometimes it can be overlooked until the onset of the paraparesis [8]. As described in other studies, all phenotypes can arise within the same family, despite identical *ABCD1 *gene mutations [8,9].

The differential diagnosis is based on the phenotype. CALD may be mistaken with psychiatric and developmental disorders, acute disseminated encephalomyelitis, multiple sclerosis, and other leukodystrophies. Conversely, myeloneuropathy’s differential diagnosis encompasses multiple sclerosis, amyotrophic lateral sclerosis, vitamin B12 deficiency, and progressive spastic paraparesis. Finally, primary adrenal insufficiency can arise from diverse causes such as congenital adrenal hyperplasia, autoimmune and infectious adrenalitis, and some drugs [2,15].

Screening male children with primary AI for X-ALD using a VLCFA panel should be considered, particularly after ruling out the most common causes or when learning difficulties are evident. Additionally, a brain MRI should be performed in those patients with AI and learning disabilities and/or recent hyperactivity diagnosis, as it is fundamental to rule out cerebral demyelination [4,10,16,17].

Adrenal screening re-assessment (every 4-6 months) in patients with X-ALD is recommended by the Pediatric Endocrine Society (2006), as an ACTH determination >100 pg/mL and cortisol < 5-10 mcg/dL are suggestive of primary AI and these findings favor the performance of a cosyntropin stimulation test. However, children with abnormal ACTH (> 300 pg/ml) and cortisol (< 18 mcg/dL) levels should begin daily and stress-dose glucocorticoid replacement without the need to previously perform a stimulation test [8,18]. 

Untreated adrenal insufficiency is potentially life-threatening. The typical manifestation of adrenal insufficiency in X-ALD involves a deficiency in glucocorticoids while sparing mineralocorticoid function. However, mineralocorticoid deficiency may also occur. Oral hydrocortisone is the first-choice replacement therapy for children (from 7.5 to 15 mg/m2/day divided into two, three, or four doses), given its short half-life, rapid peak in plasma concentration, lower potency, and fewer adverse effects compared to prednisolone and dexamethasone. If the patient exhibits normal aldosterone secretion, there is no requirement for mineralocorticoid replacement. However, if there is confirmed aldosterone deficiency, fludrocortisone should be initiated (0.1-0.2 mg/day) [15].

Despite not being available in Portugal, newborn screening for X-ALD has the potential to identify boys with AI in a timely manner to treat and prevent life-threatening adrenal crises, especially since there is a lack of a well-established genotype-phenotype correlation [4,19,20]. Confirmation of the genetic diagnosis of X-ALD enables genetic counseling, family planning, and preimplantation genetic diagnosis. The genetic study also allows the early identification of asymptomatic patients, who represent half of the sample size in some studies and can benefit the most from HSCT [8,10,17]. For most patients with X-ALD, there is currently no curative or preventive treatment. The HSCT performed at an early stage (up to 12 months after CALD symptoms’ onset) has a much better prognosis than those performed after that time. Following structural neurological damage, the possibility of stabilization and improvement of the disease is practically non-existent, and sometimes it entails a 3.9 times higher risk of death when compared to patients who undergo early HSCT [21]. The autologous HSPC for X-ALD treatment was recently approved by the European Medicines Agency, but prospective studies (namely randomized control trials) with longer follow-up periods are crucial to properly assess patients’ clinical outcomes [8,11].

## Conclusions

X-ALD is a rare genetic disorder and it is of utmost importance to early recognize its signs and symptoms and establish a definitive diagnosis in order to start a timely and multidisciplinary approach, allowing for a partial improvement in the survival and clinical outcomes of these patients. Genetic confirmation of the diagnosis is essential enabling genetic counseling and preimplantation genetic diagnosis.
